# Sharing knowledge on the treatment of childhood cancer

**DOI:** 10.2471/BLT.19.020719

**Published:** 2019-07-01

## Abstract

The Russian Federation is sharing lessons drawn from 30 years of treating childhood cancer, transferring knowledge to fellow Commonwealth of Independent States countries and beyond. Andrey Shukshin reports.

Dr Guzel Muftakhova has a difficult job. A paediatric oncologist at the Dmitry Rogachev Center in Moscow, she has the unenviable task of telling parents that their child has cancer. 

“Spotting the symptoms is notoriously difficult, as the usual presentation is non-specific,” Muftakhova says. “Often the child has been a little more tired over the last couple of weeks, not playing as much or has a few more bruises than usual. Few parents would see through it and raise the alarm”.

And raising the alarm early is crucial. Because childhood cancer generally cannot be prevented or detected through screening. The best way to improve the child’s chances of surviving cancer is to ensure early and accurate diagnosis, and then to deliver the most effective treatment.

Muftakhova’s job, and the job of her Russian peers, has become somewhat easier over the past few decades as the prognosis of the children coming to the centre has improved, reaching levels comparable to those attained in high-income countries.

“Overall paediatric cancer survival rates in the Russian Federation have risen from around 10% in the early 1990s to around 80% today,” says Professor Alexander Rumyantsev, president of the Dmitry Rogachev Center and Chief Paediatric Haematologist of the Ministry of Health.

Of the approximately 4000 children registered as having cancer in the Russian Federation annually, around 3200 will survive.

One of the reasons for that improvement is the government’s increased prioritization of paediatric cancer in the mid-2000s, the most notable expression of which being the establishment of the Dmitry Rogachev Center itself.

“The decade of political and economic turmoil that followed 1991 was a trying time for doctors in our country.”

Kirill Kirgizov

Established in 2005, the centre treats children, adolescents and young adults for cancer, haematological non-malignant disorders and immune deficiencies. 

According to Rumyantsev, over 2000 patients are admitted to the 470-bed facility every year, receiving treatments that include bone marrow transplants and CAR-T-cell therapy, in which T-cells are genetically modified to create chimeric antigen receptors (CARs) that allow the T-cells to find and destroy cancer cells when injected back into the patient.

“The work we do here has made a vital contribution to progress on paediatric cancer survival rates in our country,” says Rumyantsev.

However, as Rumyantsev himself points out, the centre is just one chapter in a story that dates back to the late 1980s, one of the early spurs to action on childhood cancer being the 1986 Chernobyl disaster. It is a story largely defined by socio-economic upheaval, including the collapse of the Soviet Union in 1991, the year the government set up the Paediatric Haematology Research Institute, the predecessor of the centre.

“The decade of political and economic turmoil that followed 1991 was a trying time for doctors in our country,” says Kirill Kirgizov, deputy director of the Institute of Management and Translational Technologies at the Dmitry Rogachev Center. “In the beginning we were literally surviving on crumbs.”

As challenging as those beginnings were, they forced the Russian Federation’s oncologists to think hard about developing treatment protocols and guidelines that were not only effective but affordable.

With a view to supporting that effort, in 1991 the Paediatric Haematology Research Institute, with the backing of the health ministry, took the decision to initiate studies to identify optimal approaches to treatment.

“The implementation of prospective multi-centre studies to optimize the treatment of malignant neoplasms in children has been key to the Russian Federation’s breakthrough in paediatric oncology,” says Rumyantsev.

Among those studies is the acute lymphoblastic leukaemia study, which was initiated in 1991 and conducted under the auspices of the Moscow–Berlin study group, a German–Russian research initiative, the German side of which was anchored at the Charité University Hospital in Berlin.

According to Professor Galina Novichkova, Director of the Dmitry Rogachev Center, prior to 1991 there was no standardized approach to treatment of acute lymphoblastic leukaemia, but in most cases, patients were hospitalized for long periods of time. “In some cases patients and their families were living in the hospital wards for months,” Novichkova says.

As a result of protracted stays in hospitals and low-quality care, many patients became infected with hepatitis B infections transmitted by contaminated blood products as well as other iatrogenic infections. To address these issues, a new protocol known as Moscow/Berlin 91 (MB-91, now referred to as MB-2015) was designed to avoid prolonged bone marrow aplasia (the disappearance of bone marrow stem cells that produce blood cells) and the resultant need for extensive supportive care, frequent blood transfusions and long hospital stays.

“After seven years of implementing MB-91, the overall survival rate was just over 70%, while all patients treated under the protocol experienced decreased toxicity,” Novichkova says, adding that the survival rate under the latest version of the protocol (MB-2015) is over 90%.

As treatment protocols and guidelines were established and published, efforts were made to ensure full implementation, and these efforts continue to this day. “All clinicians are obliged to refer to the diagnostic procedures, treatment and supportive care protocols developed by the central reference centre in Moscow,” says Kirgizov. Only the approved treatments are reimbursed by the state, encouraging adoption and adherence.

The National Society of Paediatric Haematologists and Oncologists, which was established in 2009, has oversight of the country’s 86 paediatric cancer centres and conducts regular monitoring visits to ensure quality of treatment and to provide treatment supportive care protocols to clinicians.

The Ministry of Health and the National Medical Chamber organize continuing medical education for clinicians to maintain standards and to reflect current best practice.

As a result of these initiatives, the Russian Federation has emerged as a striking success story in a region that is characterized by mixed paediatric cancer outcomes and where some countries report survival rates as low as 20%. 

According to Dr Andreas Ullrich, visiting scientist at the Charité University Hospital in Berlin and a former WHO adviser on paediatric cancer, the lack of state support reflected in a dearth of specialized clinics and specialized education and professional growth programmes are key obstacles to the development of paediatric cancer response capacity in the region.

“The similarity of health-care systems and the absence of a language barrier are perfect preconditions for efficient regional cooperation.”

Andreas Ullrich

However, Ullrich argues, one of the main reasons Commonwealth of Independent States (CIS) countries still struggle to achieve outcomes comparable to international treatment outcome benchmarks is the lack of access to the knowledge they need. 

To address this challenge, Ullrich is keen to see the lessons learned and knowledge accumulated in the Russian Federation shared with its neighbours. “The similarity of health-care systems and the absence of a language barrier are perfect preconditions for efficient regional cooperation and could be a model for other WHO regions,” he says.

Kirgizov agrees, adding that the guidelines and protocols developed in the Russian Federation could be of particular interest to countries implementing the post-Soviet model of health care.

Recent initiatives designed to encourage the transfer of knowledge related to paediatric cancer include the 10th Congress of the National Society of Childhood Haematology and Oncology, which took place in April 2019 in Sochi in the Russian Federation. The congress brought together representatives from the Dmitry Rogachev Center, the National Society of Paediatric Haematologists and Oncologists, and childhood cancer experts from CIS countries as part of efforts to achieve better outcomes for children with cancer in the CIS.

More recently, in May 2019, the Dmitry Rogachev Center and the Belarusian Research Centre for Paediatric Oncology, Haematology and Immunology together with St Jude Children’s Research Hospital in Memphis, the United States of America, hosted a regional conference in the Belarus capital of Minsk. The event brought together physicians, nurses and representatives from the Russian Federation and 11 other CIS countries as part of an active Eurasian network for childhood cancer.

The Dmitry Rogachev Center is also collaborating with the Asian division of the International Society of Paediatric Oncology, a global multidisciplinary society devoted to paediatric and adolescent cancer.

The International Society of Paediatric Oncology’s Asia Group includes health-care professionals, scientists and researchers from China, India and Viet Nam. According to Kirgizov, professional in China and India are looking at the MB-2015 protocol with a view to possible implementation. In addition, the Dmitry Rogachev Center has formed a Russian–Vietnamese paediatric oncology group. Vietnamese doctors are due to start working at the centre this year.

Ullrich is fully supportive of the collaboration. “Achieving what our colleagues in the Russian Federation have in the face of considerable resource constraints is quite remarkable,” he says.

**Figure:**
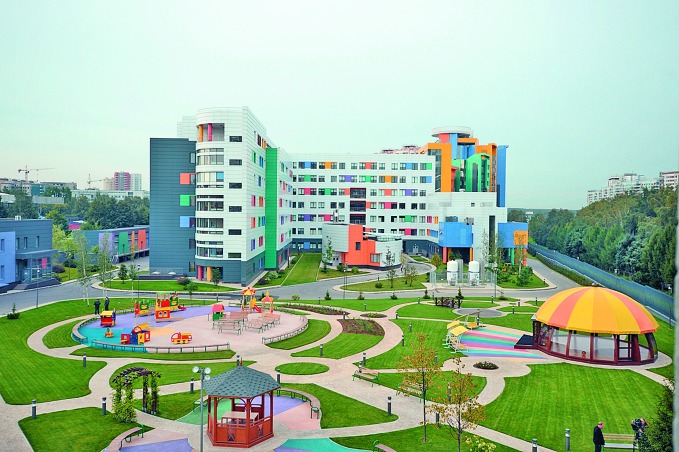
The Dmitry Rogachev Center campus, Moscow, the Russian Federation.

Courtesy of The Dmitry Rogachev Centre.

**Figure:**
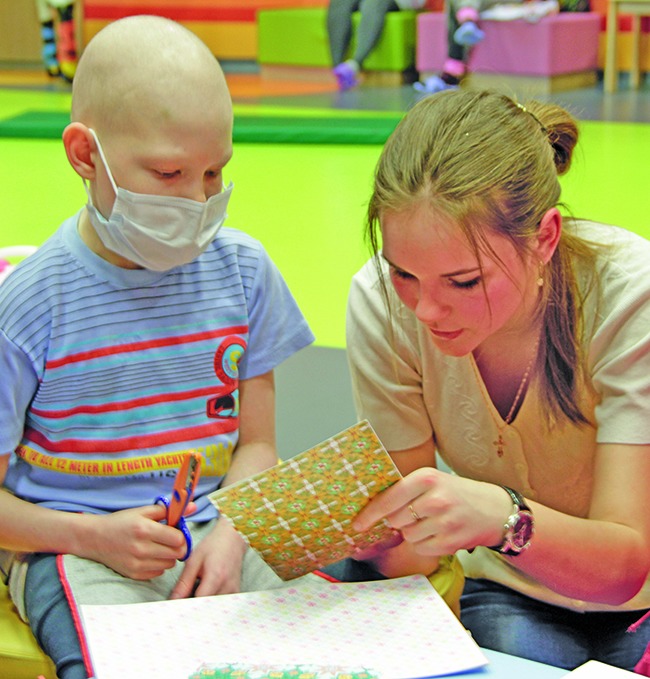
Child patients engaging in an educational activity supported by a member of staff from the Dmitry Rogachev Center**.**

Courtesy of The Dmitry Rogachev Centre.

